# Case report: Multimodality imaging revealing ruptured giant coronary artery aneurysm presenting with hemoptysis

**DOI:** 10.3389/fcvm.2023.1185089

**Published:** 2023-05-24

**Authors:** Xiangfeng Gong, Hongwei Zhang, Wanlin Peng, Gang Yang, Zhenghua Xiao

**Affiliations:** ^1^ Department of Cardiovascular Surgery, West China Hospital, Sichuan University, Chengdu, China; ^2^ Department of Radiology, West China Hospital, Sichuan University, Chengdu, China; ^3^ Department of Medical Information and Engineering, College of Biomedical Engineering, Sichuan University, Chengdu, China

**Keywords:** giant coronary artery aneurysm, hemoptysis, multimodality imaging, cardiac surgery, case report

## Abstract

Giant coronary artery aneurysm (CAA) is a relatively uncommon disease that is defined by a focal dilation of at least 20 mm and characterized by various clinical symptoms. However, cases presenting primarily with hemoptysis have not been reported. A man in his late 20 s suffering from persistent chest pain for over 2 months was transferred to our emergency department for intermittent hemoptysis lasting for 12 h. Bronchoscopy detected fresh blood in the left upper lobe bronchus without a definite bleeding source. Magnetic resonance imaging (MRI) demonstrated a heterogeneous mass and the high-intensity signals suggested active bleeding. coronary computed tomography (CT) angiography demonstrated a giant ruptured CAA wrapped in a large mediastinal mass Coronary angiography confirmed the CAA originating from the left anterior descending artery. The patient underwent an emergency sternotomy and an enormous hematoma arising from a ruptured CAA densely adhering to the left lung was identified. The patient recovered uneventfully and was discharged on the 7th day later. The ruptured CAA masquerading as hemoptysis highlights the indispensability of multimodality imaging for accurate diagnosis. Urgent surgical intervention is desirable in such life-threatening conditions.

## Introduction

Hemoptysis is a common symptom that is defined as the expectoration of blood from the respiratory tract. The severity and presentation of hemoptysis can vary widely, from mild coughing up of blood-tinged sputum to massive hemoptysis, which can lead to respiratory distress and hemorrhage shock (1, 2). The key management aspect of hemoptysis is to determine its underlying cause. It can be caused by various factors, such as infectious, pulmonary, cardiovascular, and blood disorders (1). Respiratory diseases are the most common causes of hemoptysis, but persistent hemoptysis attributed to the ruptured giant CAA has never been reported.

CAA is a rare but potentially life-threatening cardiovascular disease that progresses asymptomatically and is typically discovered incidentally during coronary angiography or coronary CT angiography. Kawasaki disease is an important cause of CAA, especially in children (3). The reported prevalence varies from 3 to 49 per 1,000 individuals with apparent male dominance and proximal coronary segment predilection (4). Giant CAA are even rarer, with an estimated incidence of approximately 0.02% (5). Patients with giant CAA may present with various clinical symptoms, including acute coronary syndrome or myocardial infarction resulting from intracavitary local thrombosis, compression of adjacent structures due to dilating vessels, and acute pericardial tamponade caused by aneurysm rupture (6). Here, we present an extremely rare case of giant CAA that manifested as intermittent hemoptysis and persistent chest pain. Through this case report, we demonstrate the potential value of multimodality imaging in diagnosing unexplained hemoptysis and improving operative procedures for active hemorrhage originating from the CAA.

## Case presentation

A 27-year-old man with episodes of hemoptysis for the past 12 h and left-sided chest dull pain without apparent cause for 2 months was referred to the emergency department. The amount of hemoptysis was approximately 200 ml, with no dizziness, tachycardia and hypotension. Initial physical examination revealed decreased breath sounds with crackles on the left side, and the chest pain was not related to breathing. Additionally, the patient had symptoms of hypoxemia and tachypnea, indicating a potential respiratory or cardiovascular condition. Troponin T and brain natriuretic peptide levels were elevated at 313.8 (reference range: 0–14 ng/L) and 506 (reference range: <88 ng/L) ng/L, respectively, while other laboratory parameters were within normal limits. The patient had no previous medical history of tuberculosis, bronchiectasis, or chest trauma, and denied any prior occurrence of Kawasaki disease or chronic inflammatory disorders. The patient initially sought medical attention at a local hospital where a CT scan was performed and was diagnosed with “pneumonia” 2 months ago. However, despite being discharged after a 9-day hospital stay, the patient continued to experience persistent chest pain symptoms.

**Figure 1:**
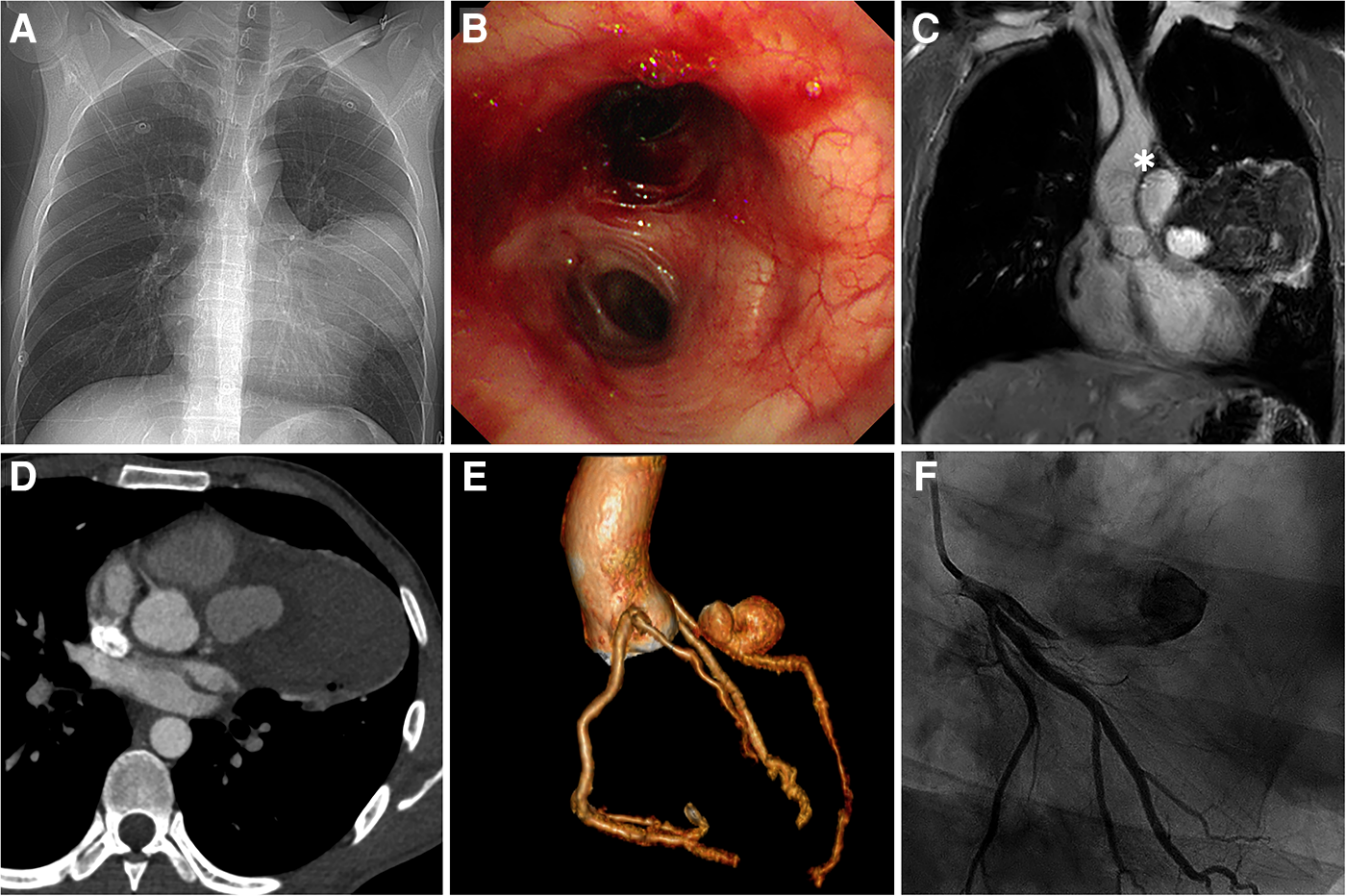
Multimodal imaging. (**A**) Anteroposterior chest x-ray. (**B**) Bronchoscopy view at the level of the left lobar bronchus. (**C**) Coronal view of thoracic MRI. (**D**) Coronary CT angiography demonstrating LAD with a large aneurysm wrapped in the hematoma (asterisk). (**E**) Preoperative coronary CT angiography: 3D reconstruction view. (**F**) Coronary angiography. LAD, left anterior descending artery.

**Figure 2:**
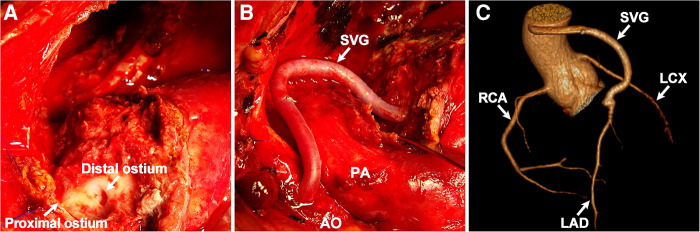
Intraoperative findings and postoperative images. (**A**) Ruptured CAA. (**B**) Coronary artery bypass surgery. (**C**) Postoperative coronary CT angiography: 3D reconstruction view. SVG, saphenous vein graft; AO, aorta; PA, pulmonary artery; RCA, right coronary artery; LCX, left circumflex artery; LAD, left anterior descending artery.

The patient was admitted for further investigation due to the unclear cause of hemoptysis and chest pain. Chest x-ray (Figure 1A) showed an enlarged left cardiac silhouette with a conspicuous bulge encroaching on the left upper-middle lung field. In addition, bronchoscopy (Figure 1B) detected fresh blood in the left upper lobe bronchus, however, the definitive bleeding source was not identified. Subsequently, thoracic MRI with a T1W1 sequence (Figure 1C) demonstrated that the heterogeneous mass was essentially caused by active bleeding, and the possible diagnosis of a mediastinal tumor was excluded. Meanwhile, coronary CT angiography (Figures 1D,E) revealed a giant suspicious left coronary artery aneurysm (37 mm × 23 mm) wrapped in a large mediastinal mass (103 mm × 75 mm), which extended to the left chest wall and infiltrated the lung lobe. Furthermore, coronary angiography (Figure 1F; Supplementary Video S1) confirmed a saccular CAA originating from the middle portion of the left anterior descending artery. Based on these findings, the potential cause of hemoptysis was considered to be CAA rupturing into the left pulmonary parenchyma. The next day, the patient underwent emergency surgery due to the concern of a potentially life-threatening massive bleeding or acute myocardial infarction caused by the complete rupture of the coronary artery aneurysm. Preoperative transesophageal echocardiography showed a pericardial mass with normal cardiac function. During the median sternotomy, the pericardium prominently protrudes to the left, thereby presenting an ill-defined margin with pleura. After the removal of the limited thrombi eroding the left pleura densely and adhering to the left upper lung, a large pseudoaneurysm arising from a ruptured left CAA was identified (Figure 2A; Supplementary Video S2). The aneurysmal sac was resected, and a saphenous vein graft was used to bypass the distal left anterior descending artery (Figure 2B). The patient recovered uneventfully with cessation of hemoptysis, and predischarge CTA 9 days after surgery showed the disappearance of the left CAA (Figure 2C).

## Discussion

Massive hemoptysis is a high-mortality clinical emergency requiring prompt localization and control of bleeding to prevent airway obstruction and subsequent asphyxia. In this case, despite bronchoscopy confirming that the airway was not occluded, the underlying cause of hemoptysis remained a diagnostic challenge, given its numerous possible etiologies. Hemoptysis is mainly caused by bronchopulmonary diseases and occasionally by cardiovascular diseases such as rheumatic heart disease with mitral stenosis and acute thoracic aortic dissection (7). Hemopericardium and hemoptysis caused by a congenital arteriovenous malformation have also been reported (8). CAA can progress without showing any symptoms, and their rupture often leads to serious cardiovascular events, but typically not hemoptysis. In this rare case, a hematoma resulting from ruptured CAA contained the rupture and limited bleeding, thus preventing an immediate catastrophic event. However, the sizable mass compressed the lung parenchyma and led to the development of a coronary artery-to-bronchial fistula, resulting in intermittent hemoptysis.

While CT scans and bronchoscopy are commonly employed for localizing bleeding and identifying the cause of hemoptysis in many patients (9), they may not be fully diagnostic in terms of identifying subtle cardiovascular abnormalities in such cases. The case of the patient with persistent chest pain symptoms after being diagnosed with pneumonia highlights the limitations of relying solely on a single imaging technique. Multimodality imaging, which combines different imaging techniques, has been shown to provide valuable information that can lead to more accurate diagnoses and more complete characterizations of diseases (10). For instance, in the case of hemoptysis related to CAA, bronchoscopy is essential for immediate airway control and isolation of the bleeding airway. Thoracic MRI can provide detailed information on thoracic structures and rule out mediastinal and thoracic tumors. Coronary angiography can assess the size, shape, location, and frequency of aneurysms, detect thrombotic occlusions and determine collateral artery formation. However, it may not provide further information in patients with mild ectasia or small fusiform aneurysms. On the other hand, coronary CT angiography has the advantage of providing a fast 3D evaluation and allowing for the analysis of both lumen composition and the vessel wall. This can lead to more effective treatment planning and improved outcomes for patients (11).

The pathogenesis of CAA is complex and multifactorial, involving genetic, inflammatory, and mechanical factors. The most common causes of CAA are atherosclerosis and Kawasaki disease (12, 13). In Kawasaki disease, inflammation of the arterial walls can cause damage to the endothelial cells that line the coronary arteries, leading to the formation of microthrombi and activation of the coagulation cascade. These microthrombi can further induce the release of pro-inflammatory cytokines, leading to a sustained inflammatory response and promoting the formation of aneurysms. Other causes of CAAs include connective tissue disorders, infections, trauma, and congenital anomalies (14). Due to the Coronavirus Disease 2019 (COVID-19) pandemic, there has been a notable rise in the incidence of Kawasaki disease accompanied by cardiovascular complications in children and young adults. In comparison to the typical age of Kawasaki disease diagnosis, affected children tend to be older and show increased cardiovascular involvement (15). As a result, healthcare providers must remain vigilant in considering the potential presence of CAA in these patients and take appropriate steps toward diagnosis and treatment (16).

The treatment options for CAAs consist of surgical, percutaneous, and medical approaches, such as angiotensin II receptor blockers, statins, antiplatelet/anticoagulant therapy (17), intravenous immunoglobulin therapy (3), and percutaneous exclusion techniques for suitable anatomy, but potential issues with reduced deliverability, restenosis, thrombosis, and occlusion of side branches must be taken into consideration (18). The surgical approach still is preferred for the large and complicated CAAs who cannot be treated percutaneously. In this case, we performed an aneurysmectomy and giant saphenous vein graft bypass grafting. It is important to note that pseudoaneurysms may develop in giant saphenous vein grafts after myocardial revascularization. In general, cardiac surgeons prefer to use the left internal mammary artery (LIMA) to bypass the left anterior descending (LAD) artery in young patients due to its clinical advantages and long-term durability (19). However, our center's experience in treating giant CAAs suggests that these patients often have heterotopic coronary arteries that are distant from the site of aneurysm removal, making it necessary to evaluate the length of graft required for coronary artery bypass surgery. In such cases, the length of LIMA may not be adequate, and harvesting the great saphenous vein for this emergency procedure is a simple and time-saving alternative.

In conclusion, we described a rare clinical manifestation of the giant ruptured CAA and underscored the indispensability of multimodality imaging for accurate diagnosis and management of hemoptysis. While the increasing use of coronary angiography, coronary CT angiography, and MRI is likely to lead to more frequent diagnoses of CAA, the management of CAA remains a clinical challenge. Therefore, future prospective comparative trials are needed to determine the most effective strategies and optimal intervention timing to prevent serious complications. Clinicians should perform a comprehensive clinical evaluation that takes into account the patient's cardiovascular risk factors, comorbidities, and the nature and anatomy of the CAA to develop a patient-specific treatment plan.

## Conclusion

For patients presenting with hemoptysis, the initial priority is to ensure that their airway is unobstructed. It is also important to consider the possibility of coronary disease if the patient is experiencing persistent chest pain. To accurate diagnosis, multimodality imaging including thoracic MRI and coronary CT angiography are essential. Additionally, it is crucial for surgeons to confirm whether the patient has any risk factors such as coronary atherosclerosis, Kawasaki disease, or COVID-19. Treatment decisions should be individualized for each patient.

## Data Availability

The original contributions presented in the study are included in the article/**Supplementary Material**, further inquiries can be directed to the corresponding author.
